# Clinical characteristics and laboratory findings of children and adolescents who were newly diagnosed with diabetes mellitus

**DOI:** 10.1186/1687-9856-2013-S1-P16

**Published:** 2013-10-03

**Authors:** You Jin Kim, Hae Sang Lee, Jin Soon Hwang

**Affiliations:** 1 Department of Pediatrics, Ajou University School of Medicine, Ajou University Hospital, Suwon, Korea

## Objective

The incidence of childhood type 2 diabetes mellitus (DM) is increasing worldwide in parallel with an increasing prevalence of childhood obesity. We investigated the type of diabetes and the clinical characteristics in the newly diagnosed diabetic children.

## Methods

Retrospective analysis of clinical characteristics was done in 145 newly diagnosed diabetic children and adolescents under 18 years of age at Ajou University Hospital in Korea from March 2003 to February 2012.

## Results

Children diagnosed with type 1 DM were 110 out of 145 (75.9%) and 35 out of 145 (24.1%) were type 2 diabetes. Mean age of onset was 10.6 ± 0.9 years and there was no seasonal variation of incidence. 36.4% of children with type 1 DM presented initially with diabetic ketoacidosis. Mean body mass index (BMI) was 16.9 ± 3.8 kg/m^2^, mean blood glucose level was 482.7 ± 214.4 mg/dL and mean glycated hemoglobin (HbA1c) level was 12.1 ± 2.28%. Positive result was revealed in 70% of the subjects with type 1 DM for antibodies to glutamic acid decarboxylase (GAD), 1.8% for islet-cell antibodies (ICA), 20.9% for insulin autoantibodies (IAA) and 75.4% showed positive results for at least one of these autoantibodies. 35 patients (24.1%) were diagnosed with type 2 diabetes. Mean age of onset of type 2 diabetes was 12.2 ± 3.4 years. 22 out of 35 (63%) subjects were diagnosed with type 2 DM in the process of evaluating the cause of obesity without any other presenting symptoms. Mean BMI was 27.1 ± 7.9 kg/m^2^, mean blood glucose level was 229.0 ± 108.8 mg/dL and mean HbA1c concentration was 9.3 ± 2.9%. 63% of the subjects diagnosed with type 2 DM had a family history of DM and 75% were either overweight or obese. Fatty liver were diagnosed in 20% patients.

## Conclusion

Although still not as common as type 1 DM among children, type 2 DM increasingly has been seen in children. Routine medical screening in obese children and adolescents or ones with other risk factors of type 2 DM should be emphasized to make early diagnosis and start management of type 2 DM to improve long-term outcomes.

